# Comparison of biannual ultrasonography and annual non-contrast liver magnetic resonance imaging as surveillance tools for hepatocellular carcinoma in patients with liver cirrhosis (MAGNUS-HCC): a study protocol

**DOI:** 10.1186/s12885-017-3819-y

**Published:** 2017-12-21

**Authors:** Hyun A Kim, Kyung Ah Kim, Joon-Il Choi, Jeong Min Lee, Chang Hee Lee, Tae Wook Kang, Young-Mi Ku, Su Lim Lee, Yang Shin Park, Jeong Hee Yoon, Seong Hyun Kim, Moon Hyung Choi

**Affiliations:** 1 Department of Radiology, St. Vincent’s Hospital, College of Medicine, The Catholic University of Korea, 93 Jungbu-daero, Paldal-gu, Suwon-si, Gyeonggi-do 16247 Republic of Korea; 2 Department of Radiology, Seoul St. Mary’s Hospital, College of Medicine, The Catholic University of Korea, 222 Banpo-daero, Seocho-gu, Seoul Republic of Korea; 3 Department of Radiology, Seoul National University Hospital, 101 Daehak-ro, Jongno-gu, Seoul Republic of Korea; 4 Department of Radiology, Korea University Guro Hospital, Korea University College of Medicine, 148 Urodong-ro, Guro-gu, Seoul Republic of Korea; 5 Department of Radiology and Center for Imaging Science, Samsung Medical Center, Sungkyunkwan University School of Medicine, 81 Irwon-ro, Gangnam-gu, Seoul Republic of Korea; 6 Department of Radiology, Uijeongbu St. Mary’s Hospital, College of Medicine, The Catholic University of Korea, 271 Cheon bo-ro, Uijeongbu, Gyeonggi-do Republic of Korea

**Keywords:** Hepatocellular carcinoma, Liver cirrhosis, Ultrasonography, Magnetic resonance imaging, Surveillance

## Abstract

**Background:**

Ultrasonography (US) is recommended as a standard surveillance tool for patients with a high risk of developing hepatocellular carcinoma (HCC). However, the low sensitivity of US for small HCC can lead to surveillance failure, resulting in advanced stage tumor presentations. For the early detection of HCC in high-risk patients and to improve survival and prognosis, a new efficient imaging tool with a high sensitivity for HCC detection is needed. The purpose of this study is to evaluate and compare the feasibility and efficacy of non-contrast magnetic resonance imaging (MRI) with US as a surveillance tool for HCC in patients with liver cirrhosis.

**Methods:**

MAGNUS-HCC is a prospective, multicenter clinical trial with a crossover design for a single arm of patients. This study was approved by six Institutional Review Boards, and informed consent was obtained from all participants. All patients will undergo liver US every 6 months and non-contrast liver MRI every 12 months during a follow-up period of 3 years. If a focal liver lesion suspected of harboring HCC is detected, dynamic liver computed tomography (CT) will be performed to confirm the diagnosis. After the last surveillance round, patients without suspicion of HCC or who are not diagnosed with HCC will be evaluated with a dynamic liver CT to exclude false-negative findings. The primary endpoint is to compare the rate of detection of HCC by US examinations performed at 6-month intervals with that of yearly non-contrast liver MRI studies during a 3-year follow-up. The secondary endpoint is the survival of the patients who developed HCC within the 3-year follow-up period.

**Discussion:**

MAGNUS-HCC is the first study to compare the feasibility of non-contrast MRI with US as a surveillance tool for the detection of HCC in high-risk patients. We anticipate that the evidence presented in this study will establish the efficacy of non-contrast MRI as a surveillance tool for HCC in high-risk patients.

**Trial registration:**

The date of trial registration (NCT02551250) in this study was September 15, 2015, and follow-up is still ongoing.

## Background

Hepatocellular carcinoma (HCC) is the fifth most common cancer worldwide and the second leading cause of cancer-related death [1]. Cirrhosis is the most significant risk factor for the development of HCC [2, 3]. The prognosis of HCC depends on the disease status at the time of diagnosis [4, 5]. Therefore, detection of HCC at an early stage is important, and the need for regular surveillance of high-risk patients is essential. Several studies have demonstrated that HCC surveillance leads to early detection, improved overall survival, and cost-effectiveness [2, 6–8].

The current guidelines for HCC surveillance recommend ultrasonography (US) as a screening imaging test [2, 9–11]. US is highly cost-effective, non-invasive, and radiation free [12]. However, in certain patient and under certain operator conditions, US exhibits a low sensitivity for tumor detection [13, 14]. Thus, US surveillance failure and/or suboptimal surveillance are the most common reasons for advanced stage tumor presentation. A better surveillance tool with an improved sensitivity and specificity is clearly needed.

Computed tomography (CT) and magnetic resonance imaging (MRI) exhibit increased sensitivity for detecting tumors compared with US [15, 16]. Though CT is excellent for detecting and diagnosing tumors, radiation exposure and the need for contrast agents limits its usefulness [17]. Contrast-enhanced MRI is the best imaging modality for tumor detection and differential diagnosis [15, 16, 18–22]. MRI has superior tissue contrast and poses no radiation hazard [21, 23, 24]. Recently, some studies have confirmed the efficacy of MRI as a surveillance modality for HCC [18, 25]. However, MRI examinations with contrast have disadvantages, such as limited accessibility, high cost, and the risk of contrast-related side effects. Nevertheless, the installation of MRI scanners has been increasing worldwide, increasing its accessibility [24]. In addition, non-contrast MRI also exhibits high sensitivity for detecting focal liver lesions [26] and is comparable to gadoxetic acid-enhanced MRI for detecting primary liver cancer [27]. If the MRI examination is performed with sufficient sequences to preserve superior tissue contrast without the use of contrast agents, the risk of contrast-related adverse events is eliminated, and both the cost and examination time can be reduced. With this in mind, non-contrast MRI is potentially a good alternative surveillance tool for HCC. To our knowledge, this is the first prospective study to evaluate the use of non-contrast MRI as an HCC surveillance tool in patients with liver cirrhosis.

The purpose of this study is to investigate the feasibility and efficacy of non-contrast MRI compared with US as a surveillance tool for the development of HCC in patients with high-risk factors. Specifically, we assessed the detection rate of HCC associated with biannual ultrasonography compared with annual non-contrast magnetic resonance imaging in patients with liver cirrhosis during a 3-year follow-up period.

## Methods/design

### Study design

**Fig. 1:**
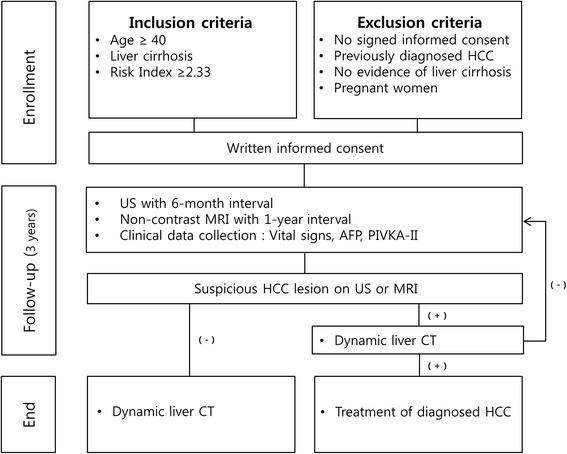
Flow chart providing an overview of the study design. US, ultrasonography; MRI, magnetic resonance imaging; AFP, α fetoprotein; PIVKA-II, protein induced by vitamin K absence/antagonist II; CT, computed tomography

This study is a prospective, multi-center clinical trial using a crossover design for a single arm of patients. Patients were recruited from second or tertiary hospitals in the Republic of Korea. Participants were ≥ 40 years of age with a diagnosis of liver cirrhosis who visited the hospital for HCC surveillance. US is performed every 6 months, and a non-contrast liver MRI is performed every 12 months without randomization. A flow chart of the study design is presented in Fig. 1.

### Ethical considerations

The study protocol was reviewed and approved by the Institutional Review Board of six participating institutions. Written informed consent was obtained from all participants by investigators. This trial was registered in an international trial registry (ClinicalTrials.gov: NCT02551250) and a domestic trial registry of the Republic of Korea (cris.nih.go.kr: KCT0001739).

### Patient recruitment and selection

Patient recruitment was performed at six participating hospitals, including the Catholic University of Korea Seoul St. Mary’s Hospital, St. Vincent’s Hospital, Uijeongbu St. Mary’s Hospital, Seoul National University Hospital, Samsung Medical Center, and Korea University Guro Hospital. Patients with a diagnosis of liver cirrhosis who visited the medical center for HCC surveillance and had no previous diagnosis of HCC were included in this study.

#### Inclusion criteria

1. Patient age at enrollment ≥40 years
2. Liver cirrhosis diagnosed by at least one histologic or non-histologic method:
  1. Liver cirrhosis determined by liver biopsy
  2. Non-histologic diagnosis of liver cirrhosis and portal hypertension
    - Typical imaging features of liver cirrhosis on radiologic examination
    - Portal hypertension: identification of splenomegaly on radiologic examinations or identification of esophageal or gastric varices on endoscopic examination
3. Risk index of HCC ≥ 2.33
  Risk index = 1.41 (if the age is 50 years or older) + 1.65 (if the prothrombin activity is ≤75%) + 0.92 (if the platelet count is ≤100 × 10^3^/mm^3^) + 0.74 (if HCV-Ab or HBsAg test is positive) [28]

#### Exclusion criteria

1. Patients who declined to participate in this study and did not sign informed consent
2. Patients with a previous diagnosis of liver cancer or other intrahepatic malignancy
3. Patients with a history of malignancy within the previous 5 years
4. Patients who are pregnant or breast-feeding

#### Sample size calculation

To calculate the number of subjects required to achieve statistical significance, the following assumptions were made:

1. A crossover design study was planned for the two groups between US and non-contrast liver MRI.
2. The incidence of cumulative hepatocellular carcinoma was assumed to be 15% for 3 years (5% per year) [28].
3. The sensitivity of non-contrast liver MRI was assumed to be 80%, and the sensitivity of US was assumed to be 60% [27, 29].
4. Type I error (α) = 5% and the power (1-β) = 80% were assumed.

A sample size of 192 patients was calculated to achieve 80% power for a difference in HCC detection of 60% for US and 80% for non-contrast liver MRI. The required sample size was 211 patients, assuming a drop-out rate of approximately 10%.

### Surveillance

#### Surveillance period and screening interval

**Fig. 2:**
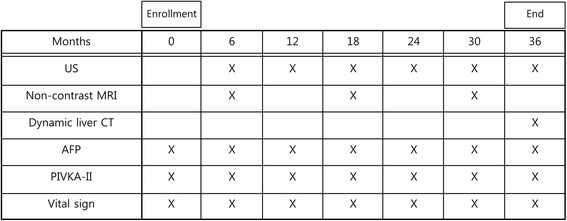
Timeline for follow-up tests. US, ultrasonography; MRI, magnetic resonance imaging; CT, computed tomography; AFP, α fetoprotein; PIVKA-II, protein induced by vitamin K absence/antagonist II

After the patients are enrolled, US for HCC screening will be performed at 6-month intervals (6 times), and non-contrast liver MRI will be obtained at the 6th, 18th, and 30th months (3 times) during the 3-year follow-up period. At the 6-, 18-, and 30-month visit, US and non-contrast liver MRI will be performed on the same day. The US is to be performed earlier by an abdominal radiologist and immediately followed by a non-contrast MRI. The laboratory investigations will include α-fetoprotein (AFP) and protein induced by vitamin K absence/antagonist II (PIVKA-II) within one month of the image study date. Vital signs obtained in the outpatient clinic will be monitored by reviewing the electronic medical record. Each patient will be followed-up for approximately 36 months. Based on the last screening round of the last enrolled patient, the total duration of this trial will be approximately 4 years. The schedule of follow-up examinations is presented in Fig. 2.

#### Surveillance tests protocol

The liver US will be performed by experienced and board-certified abdominal radiologists. The standard liver US images, as designated by the National Cancer Center of Korea, will include the following [30]: an axial scan and longitudinal scan at the branching site of a main portal vein for evaluating right and left portal veins; scans of the caudate lobe, ligamentum venosum, hepatic artery, and extrahepatic bile duct; a long axis scan of the gallbladder; an axial scan of the inferior portion of the liver for the right, middle and left hepatic veins, and intrahepatic inferior vena cava; a longitudinal scan of the left hepatic lobe; an axial scan of left hepatic lobe for the left portal vein; an axial scan of the right hepatic lobe; and an intercostal scan at posterior mid-axillary line of the liver dome and right hepatic lobe [30].

The non-contrast liver MRI will be performed without using intravenous contrast media and with 3-T MR imaging units. The scan will include fat-suppressed sequences as follows: T2-weighted fast spin echo (FSE), fat-suppressed half-Fourier acquisition single-shot turbo spin-echo (HASTE), T1-weighted gradient echo (GRE) in- and opposed-phase images, fat-suppressed T1-weighted 3D GRE (VIVE or others), diffusion weighted image (DWI) with three diffusion weightings (b = 0, 50 and 500 s/mm^2^), and afferent diffusion coefficient (ADC) maps generated using b = 0 and 500 s/mm^2^. The liver MR images will be obtained with 5 mm thickness and no gap.

#### Imaging criteria for suspicious HCC

Suspicious findings for HCC on US are defined as follows: 1) newly developed hepatic nodule 1 cm or greater in size, and 2) the nodule does not exhibit typical findings associated with a simple hepatic cyst or typical hepatic hemangioma. A hepatic nodule satisfying two US criteria will be regarded as suspicious HCC. Even if a hepatic nodule is 1 cm or larger, a lesion that was previously diagnosed as benign or exhibits minimal interval change will be excluded.

Suspicious findings on MRI suggestive of HCC are defined as follows: 1) hepatic lesion with high signal intensity on T2-weighted image (T2WI), 2) fat-containing lesion on T1-weighted image (T1WI), 3) size increase greater than 100% in >6 months, and 4) diffusion restriction on diffusion weighted image (DWI). However, a hepatic lesion with bright signal intensity on T2WI that is suggestive of a simple hepatic cyst or hepatic hemangioma as well as a lesion that was previously shown to be a benign hepatic lesion will be excluded. The MRI of the liver will be interpreted by an experienced abdominal radiologist, who will be blinded to the results of the US examination.

### Diagnostic work-up for HCC confirmation

If HCC is suspected on liver US or non-contrast liver MRI, a dynamic liver CT with 4 phases will be performed for confirmation. Imaging criteria of arterial hypervascularity and venous or delayed phase washout will be relied on to reach a diagnosis of HCC on dynamic liver CT [9]. If patients are diagnosed with HCC on dynamic liver CT, participation in the clinical trial will be terminated, and the patient will be immediately referred for treatment. If there is no evidence of HCC on dynamic liver CT, the patient will return to the regular surveillance schedule as outlined. If indeterminate lesions are identified on dynamic liver CT, liver MRI or biopsy will be considered. After the last surveillance round, patients with no suspicion of HCC or who have not been diagnosed with HCC during the 3-year follow-up period will be evaluated with a dynamic liver CT to exclude false-negative findings.

### Screening failure and drop-out criteria

Patients who have withdrawn their informed consent prior to the first examination will be regarded as a screening failure. If patients do not receive the next test within one month before or after the appropriate interval from the previous test date, they will be disqualified and labeled dropouts.

### Endpoints

Primary endpoint: comparison of the HCC detection rate by US performed at 6-month intervals with that of yearly non-contrast liver MRI during a 3-year follow-up.

Secondary endpoint: survival of the patients who developed HCC during the 3-year follow-up period.

### Data collection

Data will be collected using electronic case report form (eCRF) (cc.mediex.co.kr). Investigators or designated qualified staff in each institution will enter patient data obtained from US, MRI and laboratory tests during surveillance into eCRF. A valid username and password are needed to log into the eCRF system to secure patient data. Each patient is automatically assigned a unique identification (ID) number at the time of enrollment according to the institution and registration order.

### Statistical analysis

The sensitivity, specificity, positive predictive value, and negative predictive value of liver US and non-contrast liver MRI for the detection of HCC will be determined and compared. Detection rates and false referral rates will be evaluated. The difference in the detection rate of each modality will be compared using the McNemar test. A survival analysis of the patients who develop HCC during the study period will be determined according to the Kaplan-Meier method. A comparison of the survival curves will be made by means of the log-rank test. For continuous variables among clinical and demographic data, the mean, standard deviation, median, minimum, and maximum values will be presented. For comparison between patients with HCC or those without HCC during the study period, Student’s t-test will be used for continuous variables with a normal distribution, and Wilcoxon rank-sum test will be used for those with a non-normal distribution. A chi-square test will be used for categorical variables. Statistical analyses will be performed using SPSS, version 21.0 (IBM, New York, NY). *P-*value ≤0.05 will be considered indicative of a statistically significant difference.

## Discussion

Disease surveillance tools must be sensitive, cost-effective, and accessible. US is an efficient tool in terms of cost-effectiveness and ease of accessibility. However, US has a number of drawbacks, including limited depth penetration, poor sensitivity for detecting small lesions, and an inability to assess the entire liver [3, 20]. The efficacy of US is also operator-dependent and can be affected by several patient characteristics, such as obesity, a fatty liver, and macronodular cirrhosis [2, 13, 14, 29, 31]. All of these factors impair the sensitivity of US screening for HCC. Nevertheless, US is exclusively used as an imaging tool for HCC surveillance. A new and highly sensitive imaging modality is therefore needed for early detection of HCC in high-risk patients, which will improve survival and prognosis.

We designed a prospective crossover design with a single arm of patients. Of course, a randomized controlled trial (RCT) is the best design for comparing the effectiveness of an intervention. To date, there have been two RCTs evaluating HCC surveillance by US in China [6, 32]. However, these two studies were plagued by serious ethical concerns. There was a lack of information regarding the process of informed consent and the availability of local clinical services. In addition, participants were not offered the options of undergoing nonrandomized screening and declining screening. As a result of these improprieties, regulations surrounding research ethics have been strengthened, and there have been no RCTs evaluating HCC surveillance tools [33]. We believe that this present study design is able to properly assess the efficiency of surveillance tools for HCC.

MRI is the most sensitive modality for the detection of HCC [15]. However, MRI is not commonly used as a screening modality due to the high cost, long examination time, low accessibility and the risk of adverse effect from the contrast media. There is also little evidence supporting the efficacy of MRI for HCC surveillance. In this trial, we modified two factors to counter these objections to using MRI examination as a surveillance tool. First, the MRI will be performed without the use of intravenous contrast. Arterial enhancement after contrast injection is one of the major criteria for diagnosing HCC. However, in reality, intravenous contrast is not necessary in a surveillance step. Rather, it is more important to detect focal hepatic lesions with high sensitivity in high-risk patients. T2WIs have a high sensitivity for detecting focal liver lesions, and DWIs are effective in detecting and characterizing HCC in cirrhotic patients [34, 35], especially small HCC (<2 cm) [36]. In addition, high b-values on DWI offer excellent specificity in distinguishing HCC from benign cirrhotic nodules and pseudo-lesions [37]. If the MRI examination is performed using these basic sequences without contrast, this method is quite adequate for HCC surveillance. The study also takes only 10–15 min, which is less time than when contrast-enhanced dynamic images are obtained.

In the study protocol, MRIs will be obtained annually and US bi-annually. MRIs will be obtained every 12 months, an acceptable interval range for HCC surveillance based on the recommendation to obtain imaging studies every 6 to 12 months [15]. Thus, the high cost of MRI will be offset by fewer examinations. In addition, the increased time interval between studies will be offset by the superior sensitivity of MRI for HCC detection. In summary, fewer studies compensated by superior sensitivity can decrease the overall cost of MRI and increase its appeal as a screening tool.

This is the first study to evaluate the feasibility of non-contrast MRI compared with US as a surveillance tool for detecting HCC in high-risk patients. We expect that the results support the efficacy of non-contrast MRI as a screening tool for HCC in this patient population.

## Abbreviations

ADC: Afferent diffusion coefficient
AFP: α-fetoprotein
DWI: Diffusion-weighted image
FSE: Fast spin echo
GRE: gradient echo
HBsAg: Hepatitis B virus surface antigen
HCC: Hepatocellular carcinoma
HCV-Ab: Hepatitis C virus antibody
MRI: Magnetic resonance imaging
PIVKA-II: Vitamin K absence/antagonist II
T1WI: T1-weighted image
T2WI: T2-weighted image
US: Ultrasonography
